# Longitudinal CITE-Seq profiling of chronic lymphocytic leukemia during ibrutinib treatment: evolution of leukemic and immune cells at relapse

**DOI:** 10.1186/s40364-020-00253-w

**Published:** 2020-12-09

**Authors:** Sarah Cadot, Carine Valle, Marie Tosolini, Frederic Pont, Laetitia Largeaud, Camille Laurent, Jean Jacques Fournie, Loic Ysebaert, Anne Quillet-Mary

**Affiliations:** 1 Centre de Recherches en Cancérologie de Toulouse, INSERM UMR1037, Toulouse, France; 2 Université Toulouse III Paul-Sabatier, Toulouse, France; 3 ERL 5294 CNRS, Toulouse, France; 4 Laboratoire d’Excellence Toulouse Cancer, TOUCAN, Toulouse, France; 5 Institut Universitaire du Cancer-Oncopole de Toulouse, Toulouse, France

**Keywords:** CLL, Ibrutinib, CITESeq, Immune cells

## Abstract

**Background:**

Ibrutinib, an irreversible Bruton Tyrosine Kinase (BTK) inhibitor, has revolutionized Chronic Lymphocytic Leukemia (CLL) treatment, but resistances to ibrutinib have emerged, whether related or not to BTK mutations. Patterns of CLL evolution under ibrutinib therapy are well characterized for the leukemic cells but not for their microenvironment.

**Methods:**

Here, we addressed this question at the single cell level of both transcriptome and immune-phenotype. The PBMCs from a CLL patient were monitored during ibrutinib treatment using Cellular Indexing of Transcriptomes and Epitopes by sequencing (CITE-Seq) technology.

**Results:**

This unveiled that the short clinical relapse of this patient driven by BTK mutation is associated with intraclonal heterogeneity in B leukemic cells and up-regulation of common signaling pathways induced by ibrutinib in both B leukemic cells and immune cells. This approach also pinpointed a subset of leukemic cells present before treatment and highly enriched during progression under ibrutinib. These latter exhibit an original gene signature including up-regulated BCR, MYC-activated, and other targetable pathways. Meanwhile, although ibrutinib differentially affected the exhaustion of T lymphocytes, this treatment enhanced the T cell cytotoxicity even during disease progression.

**Conclusions:**

These results could open new alternative of therapeutic strategies for ibrutinib-refractory CLL patients, based on immunotherapy or targeting B leukemic cells themselves.

**Supplementary Information:**

The online version contains supplementary material available at 10.1186/s40364-020-00253-w.

## Background

Development of Chronic Lymphocytic Leukemia (CLL) is highly dependent on multiple parameters such as the anatomical sites of tumor cells in which B cell receptor (BCR) activation through activation of multiple kinases, and micro-environment play a crucial role in sustaining CLL survival and/or proliferation [1]. More knowledge as to the genetic landscape and mechanisms controlling the disease has led to a shift from immune-chemotherapy to targeted therapies [2]. Amongst kinase inhibitors, ibrutinib has now become a standard therapy approved in first-line (1 L) and relapsed/refractory (R/R) settings [3–5]. Ibrutinib is an irreversible BTK inhibitor and has TEC family kinase off-target effects in immune cells [6, 7]. Ibrutinib targets CLL through different mechanisms occurring mostly in tissues: induction of apoptosis, inhibition of proliferation by disruption of the BCR and NF-kB signaling pathways, impairment of adhesion and migration leading to leukemic cell egress from tissues to blood [8–13]. Ibrutinib displays a robust clinical activity regardless of recognized high-risk features (e.g. TP53 mutations), but resistances have emerged related or not to BTK mutations. Indeed, the well-reported acquired resistance mechanism driven by BTK and/or PLCγ2 mutations [14–16] is absent in early progressions [14]. Evolution patterns of CLL before and after ibrutinib therapy have been recently addressed, but, in these studies, lymphocytosis evolution patterns only concerned leukemic cells [17, 18] and must be investigated in the context of CLL microenvironment which is critical for pathogenesis.

RNA sequencing (RNA-Seq) is a powerful method to study cellular transcriptome both qualitatively and quantitatively, but provides bulk expression analysis of samples. Rendeiro et al. [19] recently applied flow cytometry, chromatin mapping and single-cell immune profiling in ibrutinib-responsive CLL patients, such as to define the dynamic of ibrutinib response. But, to our knowledge however, both leukemic and immune cells from ibrutinib-refractory patients currently remain undescribed at this single cell level.

Cellular Indexing of Transcriptomes and Epitopes by sequencing (CITE-Seq) is an ideal new technology to characterize both phenotype and transcriptome in a single-cell RNA sequencing experiment, in both leukemic and normal cells [20]. By studying the composition of the lymphocyte population using this methodology, we monitored a CLL patient during ibrutinib treatment to evaluate whether the short clinical relapse driven by BTK mutation was accompanied by intra-clonal heterogeneous features and transcriptional/phenotypical modifications in T lymphocytes, Natural Killer and B leukemic cells. This first longitudinal follow-up of CLL under ibrutinib treatment at the single cell level unveiled a striking transcriptional co-evolution patterns, of both leukemic and normal cells, induced by ibrutinib.

## Methods

### Patient and treatment

Peripheral blood samples from an ibrutinib-treated CLL patient (420 mg/day) were obtained from the Hematology Department with informed consent and referenced in the INSERM cell bank. According to French law, the INSERM cell bank has been registered with the Ministry of Higher Education and Research (DC-2013-1903) after being approved by an ethic committee (Comité de Protection des Personnes Sud-Ouest et Outremer II). Clinical and Biological annotations of the samples have been reported to the Comité National Informatique et Liberté (the Data Processing and Liberties National Comittee). Cells were collected at 3 different time points of ibrutinib-treatment: before treatment (M0), at clinical response (3 months of treatment, M3), during progressive disease under treatment (27 months, M27).

### CITE-Seq antibodies

The following human Total-seq Biolegend antibodies were used for the CITE-Seq of PBMC: CD19 (cat: 302259), CD5 (cat: 300635), CD3 (cat: 300475), CD4 (cat: 300563), CD56 (cat: 392421), CD8a (cat: 344751), CD14 (cat: 301855), CD69 (cat: 310947), CD279(PD-1) (cat:329955), CD49d (cat: 96538), CD20 (cat: 302359), control isotype IgG1(cat: 400199).

### Flow cytometry

PBMCs were immune-stained with BV605-CD19, PC7-CD5, PB-CD3, PC5-CD4, PE-CD8, PC7-CD56, FITC-CD279 (PD-1), APC-CD20, AF421-CD49d (Biolegend) and PE-CD69 (Beckman Coulter). Samples were measured using a BD LSR II cytometer (from BD biosciences) and analyzed with BD FACS Diva software (BD Bioscience). FACS analysis was performed in parallel to CITE-Seq experiments to ensure the conservation of epitopes between fresh and frozen cells.

### CITE-Seq experiment

Cells from the different samples were thawed using a method preserving cell surface epitopes. For each sample, cells were processed according to the manufacturer’s instructions. Briefly, 2 × 10^6^ cells from each sample were numerated by trypan blue exclusion, resuspended in staining buffer with Fc blocking reagent and labeled with Total-Seq antibody-pool using 1 μg of each antibody (BioLegend). Cells were washed, filtered and resuspended at 1000 cells/μL. For each sample, the same number of cells (*n* = 13,000) was injected for CITE-Seq.

As previously described [21], Single-cell libraries (mRNA and ADT) were generated using the Chromium Controller Single-Cell instrument and Chromium Single Cell 3′ Library & Gel Bead Kit v2 and A Chip Kit and i7 Multiplex Lit (10X Genomics, with some modifications for ADT) at the CRCT Platform. The libraries were sequenced on a NextSeq500 (Illumina) in pair end sequencing 26 bp (read 1) × 98 bp (read 2) and a single index 8 bp. Gene counts were computed with Cell Ranger 3.0 using the human genome GRCh38 and then loaded in a R session with the Seurat 3.0 toolkit package as described [22]. Antibodies for CITE-Seq were counted using CITE-Seq-counter [23]. Samples were individually filtered using UMI and percentage of mitochondrial genes criteria. Genes were normalized using the sctransform package [24]. Geneset enrichment score were computed for each single cell by Single-Cell Signature Scorer [22] using genesets from MSigDB [25, 26] and visualized using Single-Cell Signature Explorer [22]. Selection of enriched genes or gene scores were defined using *p* value adjusted for multiple correction using Benjamini Hochberg (pBH) < 0.001 and a fold change (fc) > 4 when comparing two or multiple conditions.

### In vitro blood cell depletion assay

In vitro ibrutinib or venetoclax sensitivities were quantified using B cell depletion assay as previously described [27]. Briefly at each time of ibrutinib treatment fresh PBMCs were seeded at 10 × 10^6^ cells/mL in culture medium (providing long-term viability) and treated by relevant doses of ibrutinib (0.25 μM) or venetoclax (0.5 nM) for 7 days. CD19^+^/CD5^+^ (B-leukemic cells) levels were determined by flow cytometry. For each condition, absolute number of remaining B cells = total viable cell number (trypan blue exclusion determination) x % of viable CD19^+^/CD5^+^ lymphocytes (flow cytometry determination). Specific percentage of remaining B cells in treated samples = (Absolute number in treated samples/Absolute number in untreated samples) × 100. Then, specific B-leukemic cell depletion was calculated as follow: 100 - specific % of remaining B cells.

### Statistics

Mean of gene signature scores were obtained by calculating gene signature score for each single cell in each cellular population at the different time-points. Statistical analyses were performed using two-tailed Mann-Whitney test (*p < 0.05; **p < 0.01; ***p < 0.005; ****p < 0.001).

## Results

### Ibrutinib induces changes in whole blood cell patterns overtime

**Fig. 1:**
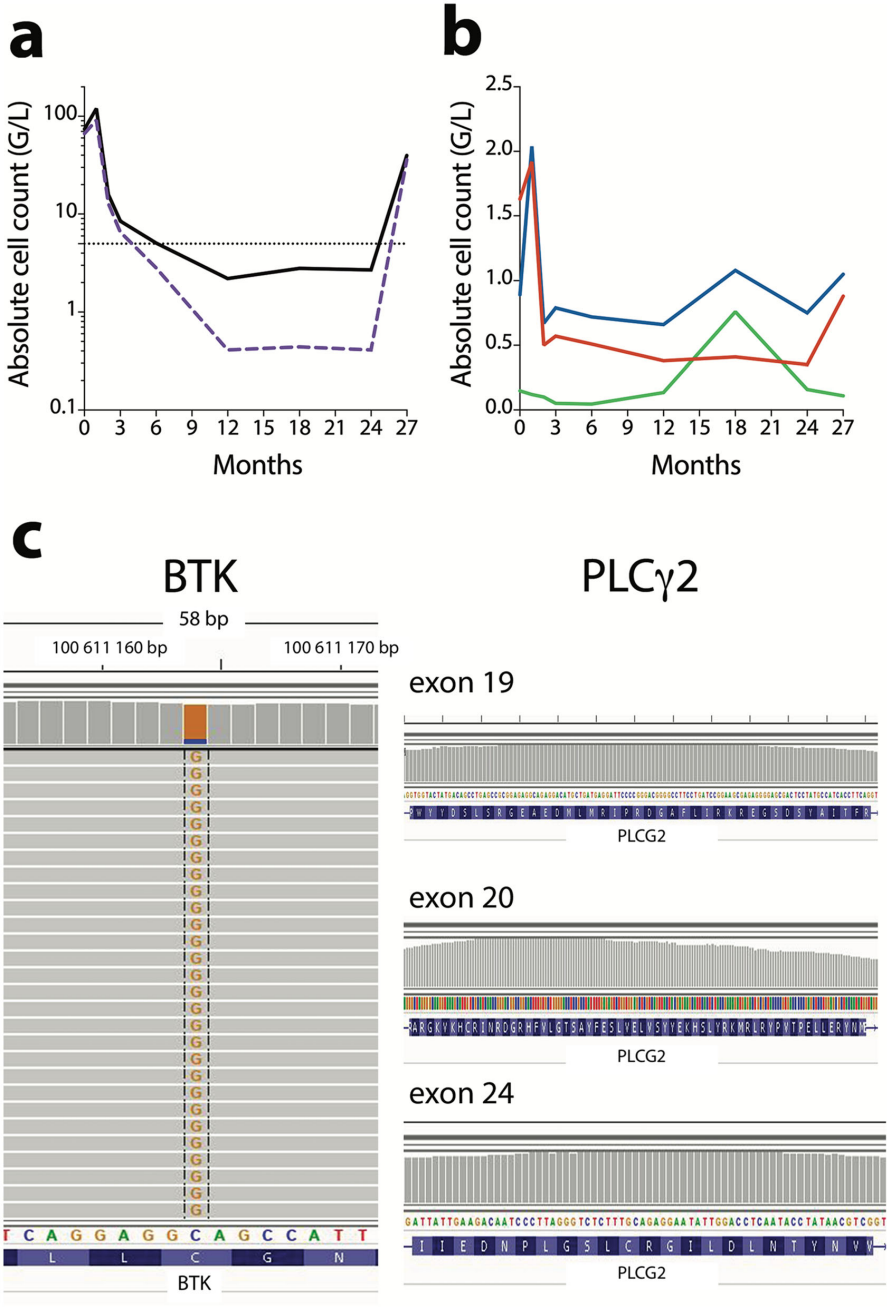
Ibrutinib treatment monitoring. **a**: Lymphocytes (), B leukemic cells (); **b**: CD4 T lymphocytes (), CD8 T lymphocytes (), Natural Killer cells (); **c** BTK and PLCγ2 mutational profile at M27

In order to assess the long-term effect of ibrutinib in CLL, we followed one ibrutinib-treated patient whose clinical features are described in Additional Table S1. Ibrutinib efficacy was monitored by absolute lymphocyte count before and during ibrutinib treatment (Fig. 1a, b). B leukemic cells and T lymphocytes were drastically reduced after 3 months of treatment whereas Natural Killer (NK) cells remained stable. Long-term follow-up (M27) showed a massive increase in B leukemic cells highlighting progression under ibrutinib. In parallel, we observed a slight increase in T lymphocytes and a decrease in NK cells. Targeted Next Generation Sequencing analysis showed BTK mutation (87%) but no PLCγ2 mutation only at the time of progression (M27) (Fig. 1c).

### ADT-labeling is required to define cellular composition

**Fig. 2:**
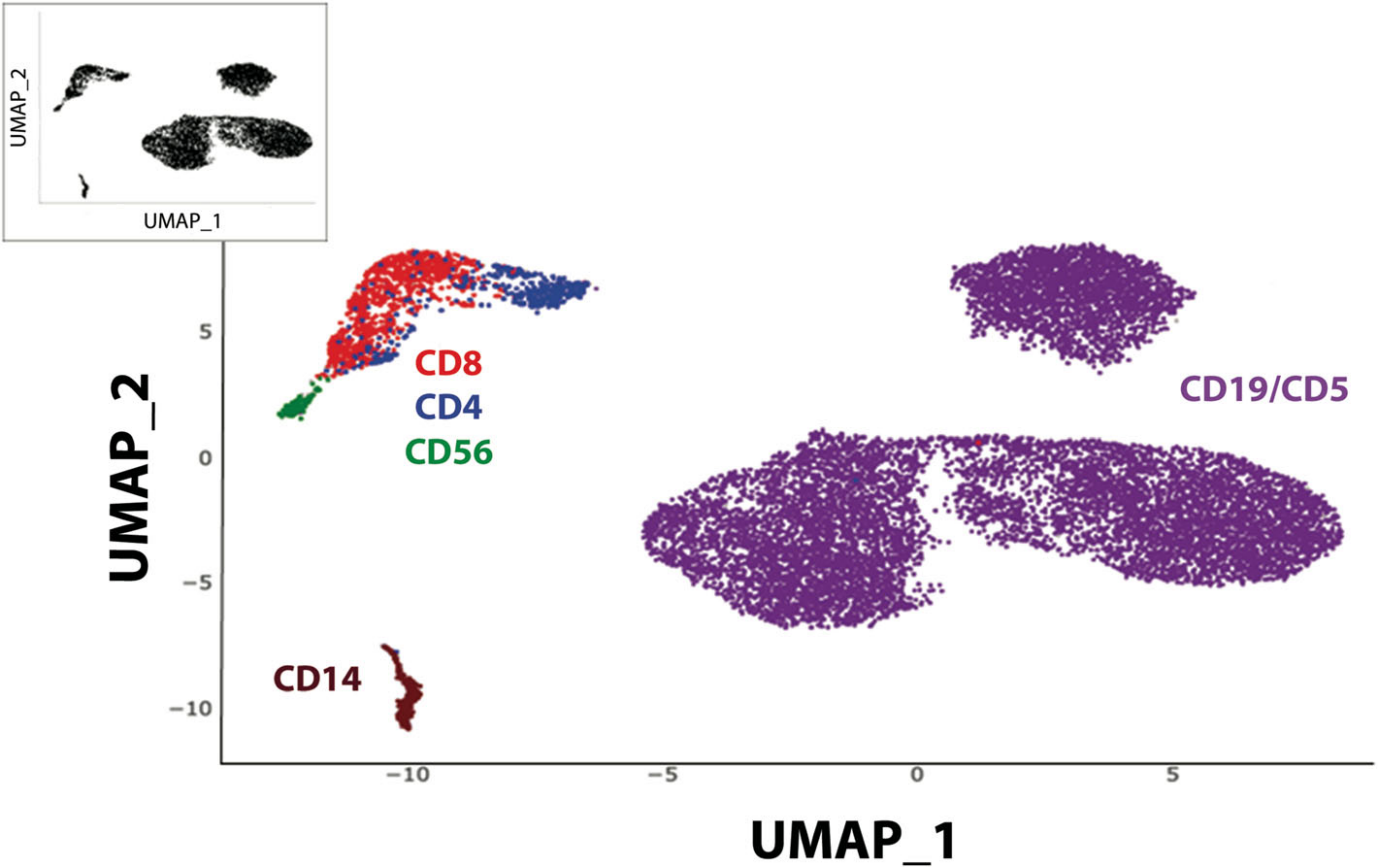
UMAP representation and identification of cellular subsets by ADT-labeling of the integrated CITE-Seq from a CLL patient during ibrutinib treatment

Blood samples were collected at M0 (before treatment), at M3 (response-time) and at M27 (time of progression) and analyzed by single cell RNA-Seq using the CITE-Seq technology. For each time-point, the same number of cells was applied in CITE-Seq. Quality control of sequencing showed comparable data for each sample: (M0: 4468 cells, reads/cell: 117261, median genes per cell: 799; M3: 3975 cells, reads/cell: 109979, median genes per cell: 683; M27: 5288 cells, reads/cell: 87955, median genes per cell: 744). The genes per cell matrix (GSE152469) were computed with Cell Ranger 3.0 and then loaded in an R session with the Seurat 3.0 toolkit package as described [21] involving the normalization, variance stabilization package sctransform and production of UMAP representation which is composed of 5 main clusters (Fig. 2 Insert). Cell types composing each cluster were identified by Antibody-Derived-Tag (ADT) cell surface labeling with specific lineage ADT-antibodies and visualized using Single-Cell Virtual Cytometer [22]. In this UMAP (Fig. 2), global composition of PBMCs revealed CD4 T lymphocytes (CD3^+^/CD4^+^; *n* = 372), CD8 T lymphocytes (CD3^+^/CD8^+^; *n* = 863), NK cells (CD3^−^/CD56^+^; *n* = 75), Monocytes (CD14^+^; *n* = 193) and B leukemic cells (CD19^+^/CD5^+^; *n* = 11,324). This analysis showed that T lymphocytes, NK cells, monocytes and B leukemic cells formed well separated clusters on UMAP. It should be underlined that B leukemic cells appeared as 3 different entities. Cellular subsets (T lymphocytes, NK, B leukemic cells) were compared using classical flow cytometry analyses and ADT-labeling. In both experiments, a similar percentage of each cellular subset was obtained (*R*^*2*^ *= 0.9894*). CITE-Seq revealed discrepancies between RNA and protein detection. Whereas CD3 was well identified by both RNA expression and ADT-labeling, CD4, as previously described [20], was only detected by ADT-labeling. CD19 or CD69 protein expression was more easily detected at the cell surface (ADT-labeling) than by mRNA expression (Additional Fig. S1). Thus, the combination of ADT-labeling with RNA expression allowed the unambiguous identification of cell populations.

### CITE-Seq analysis reveals cluster heterogeneity under ibrutinib treatment

**Fig. 3:**
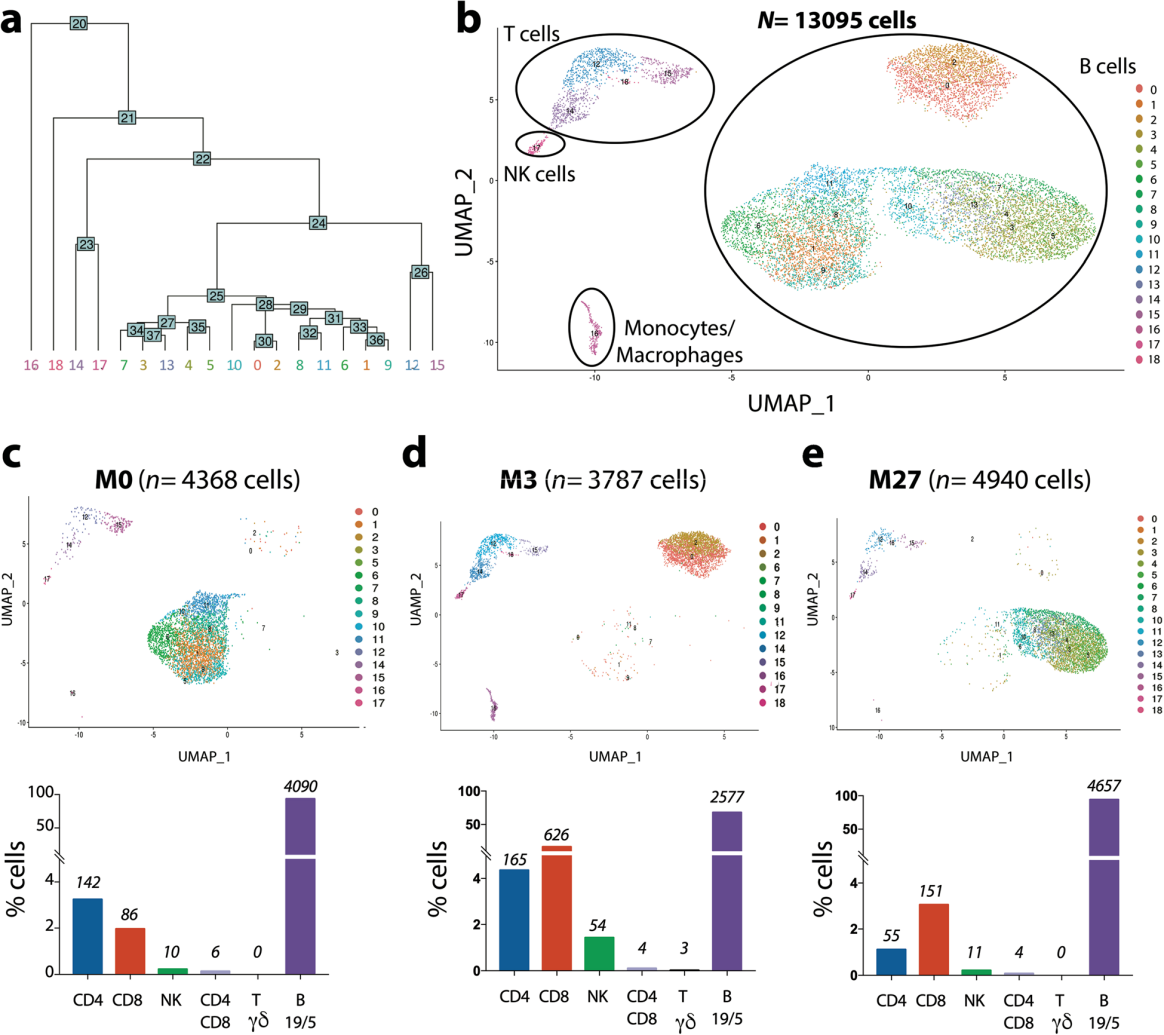
CITE-Seq clustering analysis according to time-point sampling. **a** PCA analysis unsupervised clustering; **b** UMAP representation of clusters; **c**, **d**, **e** UMAP representation and quantification of cellular populations (% cells with absolute numbers above bars) before treatment (M0) (**c**), at response-time (M3) (**d**); during progression (M27) (**e**)

Unsupervised PCA analysis delineated different clusters in the whole population under ibrutinib treatment (Fig. 3a). This clustering illustrated the micro-environment diversity and heterogeneity of B leukemic cells. First, T lymphocytes, NK cells and Monocytes were distinct but well co-localized in the three samples (M0, M3, M27) which highlights their similarity during ibrutinib treatment (Fig. 3b). Each sub-population presented the same transcriptomic profile over time despite modifications in their cell count (Fig. 3c, d, e). Deeper analysis using Single Cell Signature Explorer [22] allowed the identification and quantification of sub-populations such as the CD4^+^/CD8^+^ (*n* = 14) and Tγδ lymphocytes (cluster 18; *n* = 3)). B leukemic cells were dispatched in 3 main clusters comprising 4090 cells, 2577 cells and 4657 cells at M0, M3 and M27 respectively. Each cluster included different sub-clusters suggesting intra-clonal heterogeneity despite transcriptomic and phenotypic similarity (Fig. 3b). Independent analyses revealed that these 3 B leukemic cell clusters segregated according to treatment time (Fig. 3c, d, e).

### Ibrutinib treatment up-regulates genes in B leukemic cells and immune cells

**Fig. 4:**
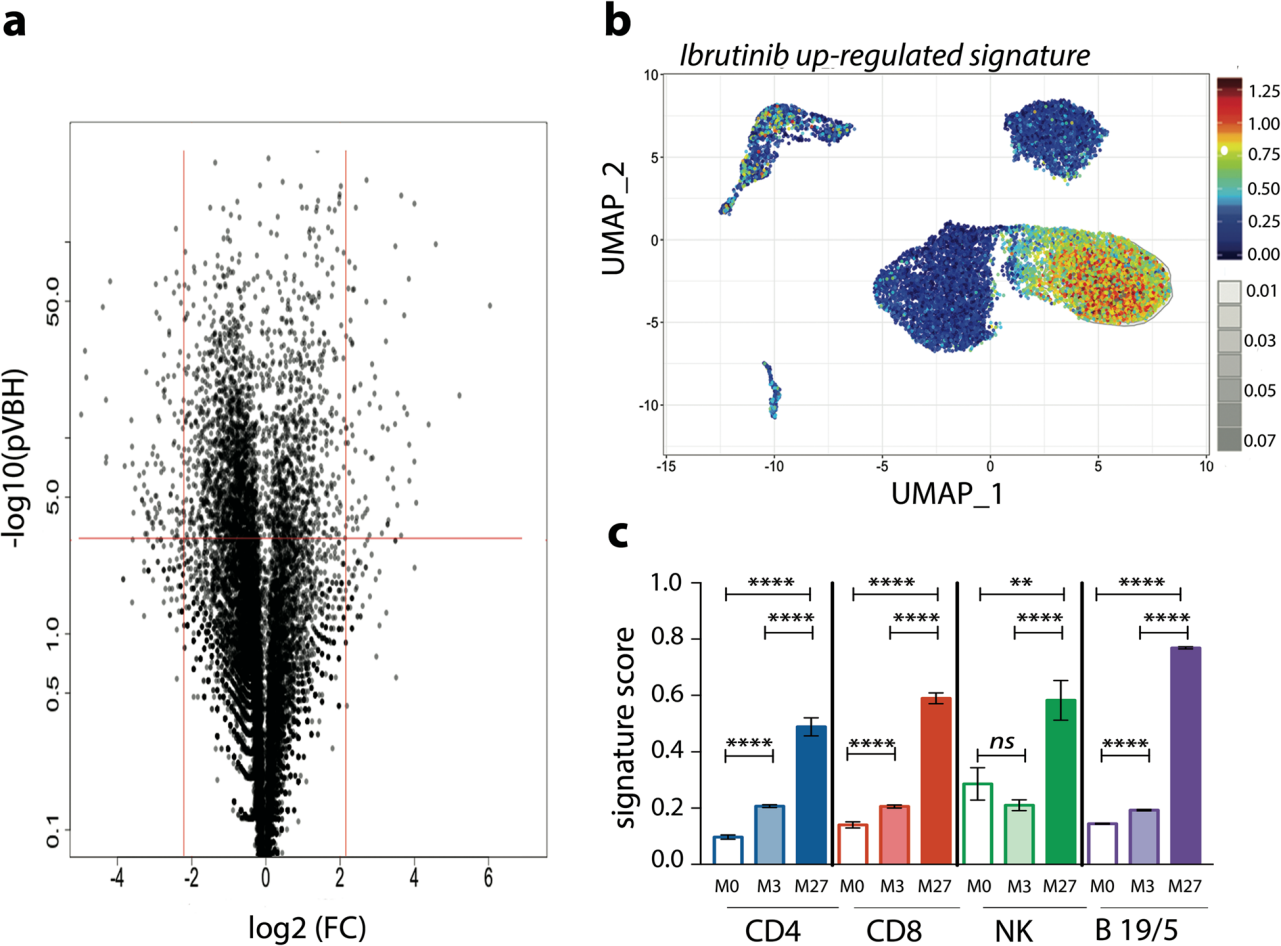
Ibrutinib up-regulated gene score. **a** Volcano plot of gene expression in all cells (M27 compared to M0); **b** UMAP representation of ibrutinib up-regulated-gene score in all cells **c** Ibrutinib up-regulated-gene score according to each cellular population and time-point sampling (mean ± SD)

Each different cluster from all cells was compared for DEGs (Differential Expressed Genes) and enrichment of gene signatures [23]. These analyses revealed some down- and up-regulated genes under ibrutinib treatment (Fig. 4a). We then compiled up-regulated genes (Table 1) to establish an “ibrutinib-up-regulated gene signature”. Some of these genes such as *CXCR4, RGS1, RGS2* were previously known to be involved in CLL pathogenesis and/or lymphocyte migration [9]. UMAP representation showed an up-regulation of these genes in all cellular subsets but more clearly in B leukemic cells at the time of progressive disease (M27) (Fig. 4b). Since normal immune cells remained co-clustered at all time-points, we combined both ADT-labeling identification and transcriptomic data to establish the ibrutinib-up-regulated gene signature to determine the mean score in each cellular population at the different time-points. This score was significantly enhanced at M3 and massively increased at M27 *i.e* at disease progression (Fig. 4c). These results suggest that similar signaling pathways were impacted by ibrutinib in both B leukemic cells and in T/NK lymphocytes.

**Table 1:** Ibrutinib up-regulated gene signature shared by immune and leukemic cells

Ibrutinib up-regulated gene signature	
*CCL4*	
*CD69*	
*CNOT1*	
*CXCR4*	
*DDIT4*	
*DUSP1*	
*JUND*	
*KLF6*	
*NFKBIA*	
*PMAIP1*	
*RGS1*	
*RGS2*	

### Ibrutinib exposure regulates genes and phenotypic markers in B leukemic cells

**Fig. 5:**
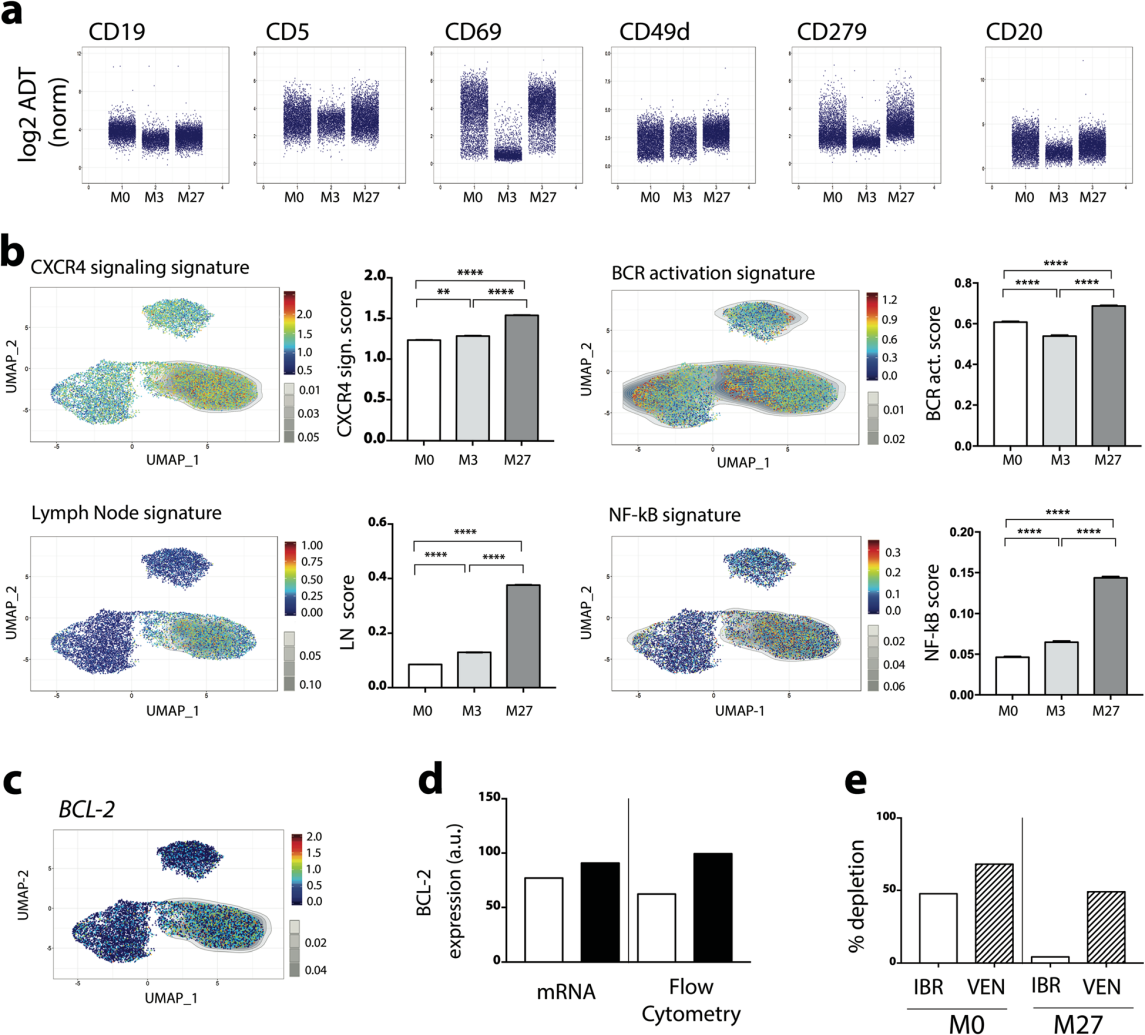
Ibrutinib-induced changes in B leukemic cells. **a** Individual CD expression; **b** UMAP representation and quantification of genes signatures; **c** UMAP representation of *BCL-2* expression; **d** Semi-quantification of BCL-2: M0 (), M27 (); **e** In vitro B cell depletion induced by ibrutinib (IBR) or venetoclax (VEN) before treatment (M0) and during progression (M27)

Changes in gene expression profiles as well as in phenotypic markers induced by BTK inhibition in B leukemic cells were assessed during ibrutinib treatment. As shown in Fig. 5a, immunophenotyping of B leukemic cells (ADT-labeling) was affected by ibrutinib with a decrease in CD19 expression even in progressive disease, whereas CD5 expression was maintained. CD69 is an activation marker, and, in CLL, an independent prognostic factor correlated to clinical progression [28, 29]. CD69^+^ CLL cells are efficiently targeted by ibrutinib in vitro [30]*.* Here, ibrutinib treatment led to a strong decrease in CD69 expression at 3 months post-treatment, followed by its re-expression correlating with progressive disease at M27 (Fig. 5a). CD49d is one of the most relevant biological predictors of overall survival and progression-free survival in CLL. Its expression decreases after short-term ibrutinib therapy [31] correlating with a reduction of CD49d-dependent pro-survival signals in lymphoid organs [32]. Here, CD49d expression increased after long-term ibrutinib treatment, suggesting a poor outcome for the patient (Fig. 5a). Finally, cell surface expression of CD279 (PD1) and CD20 markers were markedly reduced during ibrutinib response, but re-expressed during progression (Fig. 5a). Compared to M0, some genes (comprising genes up-regulated also in immune cells, Table 1) were up-regulated at relapse (Additional Table S2). Since single cell analyses of single genes do not detect all the genes in all the cells [22], the regulation of B leukemic cell signaling pathways during ibrutinib treatment was determined by signature scores (Additional Table S3) that are more reliable than genes alone. These scores were visualized using UMAP representation. The most significantly shared signatures in B CLL cells were related to CXCR4 signaling [33], BCR activation, Lymph Node- and NF-κB- signatures (Fig. 5b), i.e. signatures highly correlated to CLL pathogenesis [34]. This showed that in progressive disease under ibrutinib (M27), all signaling pathways were significantly up-regulated suggesting that resistance mechanisms such as BTK mutation were induced. Furthermore, NGS analysis revealed that in our case 87% of B leukemic cells were BTK-mutated at M27 (Fig. 1c). Our results confirmed that long-term exposure to ibrutinib led to an increase of the anti-apoptotic marker BCL-2 at both gene and protein levels (Fig. 5c, d). Resistance to ibrutinib often influences the therapeutic switch to venetoclax treatment targeting BCL-2 [35]. In an in vitro depletion assay, ibrutinib or venetoclax efficacy was tested on fresh PBMCs from our CLL patient collected before treatment and during progression. This revealed that B leukemic cells were resistant to ibrutinib at M27 but remained sensitive to venetoclax (Fig. 5e).

### CITE-Seq analysis unveils B leukemic cells heterogeneity

**Fig. 6:**
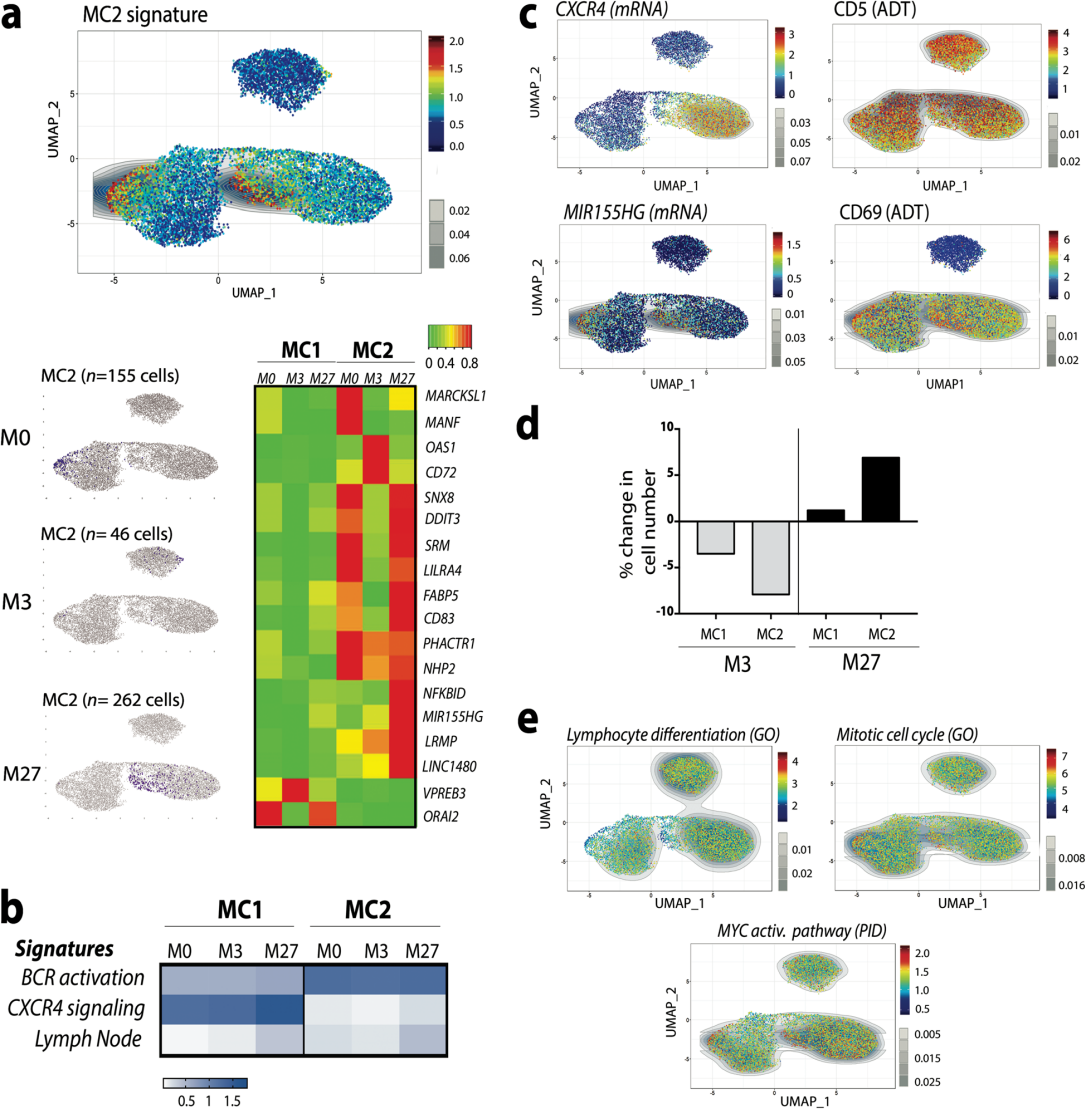
B leukemic cell heterogeneity. **a** UMAP representations of MC2 cells and heatmap of MC2 signature; **b** Comparison of ibrutinib-regulated B cell specific signatures in MC1 and MC2 cells; **c** UMAP representation of *CXCR4, MIR155HG,* CD5 and CD69 expression; **d** Fold change in cell number in MC1 and MC2 populations during ibrutinib treatment (compared to M0); **e** UMAP representation of differential signatures between MC1 and MC2 populations

Unsupervised clustering analyses (Fig. 3a, b) revealed the heterogeneity of B leukemic cells consistent with different signaling pathway signatures (Fig. 5b). By using Single Cell Signature analysis [22], we defined a signature identifying a B leukemic sub-population (Metacells 2, MC2) significantly different from all the other CLL cells, referred to below as MC1 (Metacells 1) during ibrutinib treatment (Fig. 6a). Before treatment, in contrast to MC1, the BCR activation signature [34] was up-regulated in MC2 whereas CXCR4 signaling and Lymph node signatures [33, 34] were down-regulated (Fig. 6b). Furthermore, MC2 were present before treatment and presented a lymph node cell signature associated with a down-regulation of *CXCR4* and an up-regulation of *MIR155HG* gene expression [36] and a high level of CD5 protein expression (Fig. 6c). Moreover, the ADT-based phenotyping revealed CD69 over-expression in MC2 cells (Fig. 6c). Altogether, these data suggest that the MC2 sub-population reflects prolonged circulating activated lymph node cells. Despite variability in signaling pathways between MC1 and MC2 before treatment, ibrutinib exposure increased these signatures in all leukemic cells (Fig. 6b). Time course analysis of each B-CLL sub-population revealed that MC1 and MC2 decreased at response time (M3) but re-emerged during progression (M27) with a massive increase of MC2 relative to MC1 (Fig. 6d). In order to characterize this MC2 population, its cells were analyzed for described signatures. This comparison revealed that in relation to MC1 cells, the MC2 population displayed a lower differentiation signature, together with increased expression of MYC targets and mitotic cell genes (Fig. 6e) together with stronger metabolic signatures (Additional Fig. S2) regardless of ibrutinib treatment.

### Ibrutinib regulates signaling pathways in immune cells

**Fig. 7:**
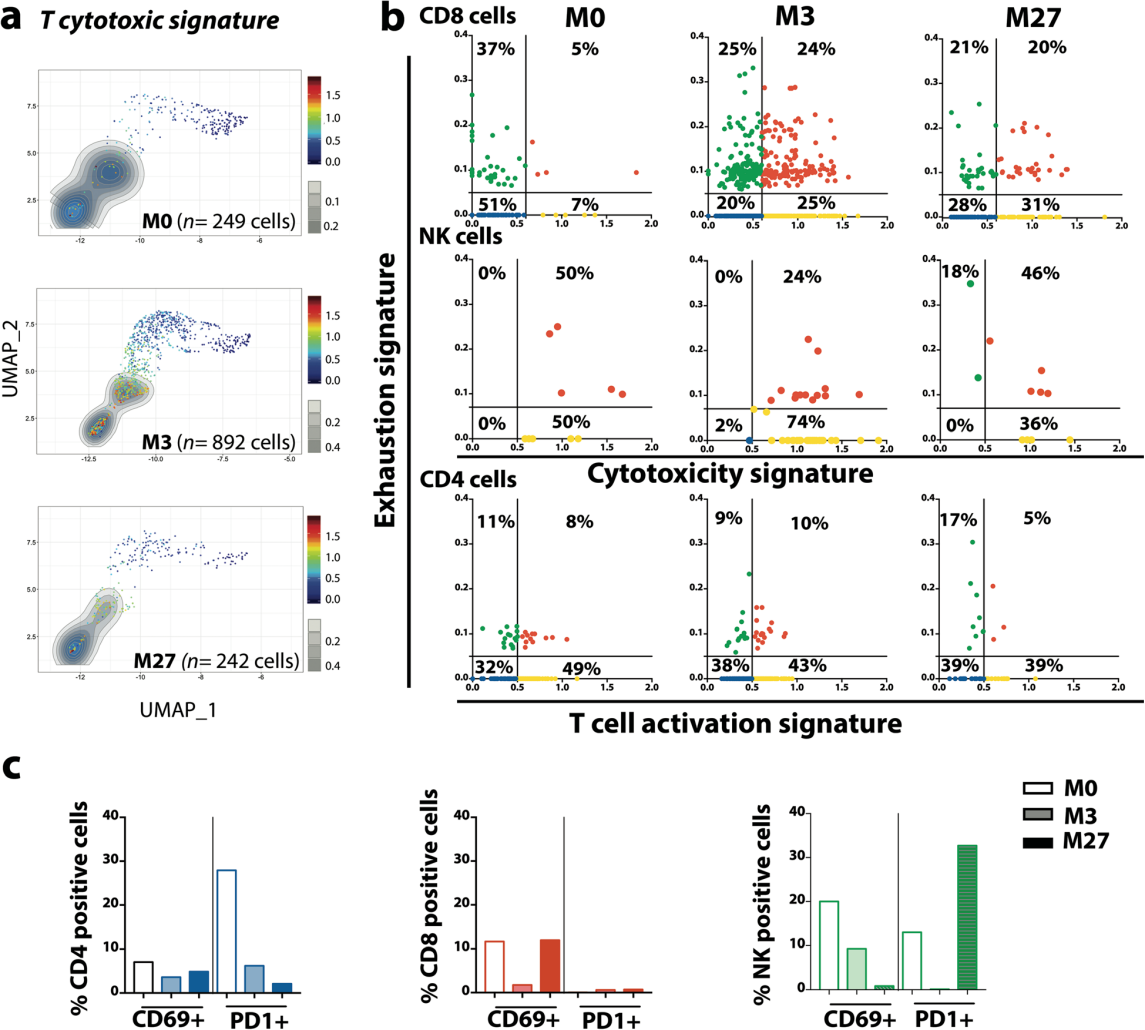
Ibrutinib-regulated pathways in immune cells. **a** UMAP representation of T cytotoxic signature in immune cells under ibrutinib treatment. **b** Cell quantification according to cytotoxic, activation and exhaustion gene scores **c** Percent of CD69 or PD1 expressing cells quantified by ADT-labeling

Ibrutinib specifically targets BTK in B cells but also the IL-2-inducible T cell kinase (ITK) expressed in T lymphocytes and NK cells [6, 7]. We then focused our single cell analysis on gated T and NK cells alone (as identified in Fig. 2) during ibrutinib treatment by quantifying specific T signatures (Additional Table S2) in each cell. Time-point analysis of T cytotoxic signature showed that ibrutinib significantly increased the expression of cytotoxic genes in CD8 T lymphocytes and NK cells (Fig. 7a). Combination of ADT-labeling identification and transcriptomic data was used to further assess T cell exhaustion and activation [22, 23, 37] (Additional Table S2) at the different time-points. This showed that during treatment, ibrutinib increased the percentage of CD8^+^ cells exhibiting a cytotoxic and/or exhaustion status. In NK cells, these cytotoxic and exhaustion scores were up-regulated at response time but decreased during disease progression. In contrast, in CD4^+^ cells, activation and exhaustion scores were not influenced by ibrutinib treatment (Fig. 7b). In parallel using CD69 (activation) or PD1 (exhaustion) ADT-labeling, we quantified activated (CD69^+^) or exhausted (PD1^+^) immune cells (Fig. 7c). Despite an up-regulation of exhaustion gene score, the percentage of CD4 and CD8 expressing PD1 decreased under ibrutinib exposure suggesting that PD1 alone did not reflect the entire transcriptomic profile of exhaustion status. In contrast, exhaustion gene score and the percentage of NK PD1^+^ increased during progression, consistent with the deregulation of cytotoxic NK cells (Fig. 7c). Thus, new therapeutic strategies must take into account the ibrutinib modulation of cytotoxicity, activation and exhaustion of immune cells.

## Discussion

Genomic, transcriptomic, proteomic analyses and flow cytometry are common technologies used to define more efficient clinical strategies for CLL. Emerging methods based on single cell analysis provide further knowledge on CLL heterogeneity. Nevertheless, these new techniques are often used on purified leukemic cells and do not allow the analysis of the microenvironment within the same sample. CITE-Seq is a new powerful method to analyze both cellular proteins and transcriptome at single cell level. Using this new technology, we report here that ibrutinib treatment biases gene expression and signaling pathways during the therapeutic response or progression not only in leukemic cells but also in the normal immune cell population. Our analyses confirmed heterogeneity of CLL and identified different biological pathways in these leukemic sub-populations. At first, ADT-labeling enhanced the identification of each cellular population present in the samples and we cleared further mis-interpretation using single cell RNAseq approaches. Of note, while UMAP clustering revealed massive differences in B leukemic cells during ibrutinib treatment, normal PBMCs (T lymphocytes, NK, and monocytes) clustering was not affected, showing that normal PBMC transcriptomes are less impacted by ibrutinib exposure compared to B leukemic cells.

Our analyses focused on the B leukemic cells, highlight the regulation of genes and transcriptional pathways during ibrutinib treatment and therapy escape. Here, resistance to ibrutinib correlated with an up-regulation of genes involved in key signaling pathways of CLL, such as CXCR4 or BCR activation, and NF-κB signature and Lymph Node features, but also in trafficking, migration, homing or cellular exhaustion. Individual gene analyses under ibrutinib exposure revealed continuous or transient gene regulation that was not related to protein expression. For example, the *PDCD1* gene was always down-regulated by ibrutinib whereas CD279 (PD1) surface expression was down-modulated at response time and re-expressed during progression. This suggests that PD1 expression in B leukemic cells can be used as a marker of progressive disease. Another important target in CLL is the anti-apoptotic protein BCL2, which is notably over-expressed in patients resistant to ibrutinib [38]. Both gene expression and protein analyses confirmed that ibrutinib induced a continuous increase of *BCL-2.* In addition, up-regulation of the *MS4A1* gene related to the increase of CD20 expression in ibrutinib-resistant cells highlights the benefit of a therapeutic switch to venetoclax associated with anti-CD20 antibodies [39].

CITE-Seq analyses revealed heterogeneity in B leukemic cells in terms of ibrutinib response. While the majority of cells presented common features, a sub-population (MC2) exhibited an inverse gene expression profile, despite a common up-regulation of signaling pathways under ibrutinib. Indeed, BCR signaling was highly up-regulated before treatment in MC2 cells, whereas the CXCR4 pathway was down-regulated. Moreover, MC2 cells presented lymph node features such as over-expression of CD5, CD69, *MIR155HG*, a lymph node activation signature, and a less marked differentiation status. In addition, MC2 cells were highly enriched during progression suggesting that these cells could have a more “aggressive” potential.

Interesting features of MC2 cells are up-regulation of Myc-targeted genes, signaling pathways related to mitochondrial processes and DNA metabolism as well as down-regulation of genes involved in mitotic cell cycle checkpoints. c-Myc plays a role in cell cycle progression, but much of its biology is involved in enhancing cell growth and metabolism [40]. Targeting Myc transcriptional regulation is currently evaluated in cancer clinical trials using BET protein bromodomain antagonists. In mantle cell lymphoma synergistic activity of BET antagonists combined with a Bcl-2 inhibitor has been demonstrated in cells resistant to ibrutinib [41]. BRD4, one of the members of the BET protein family, is often required for Myc expression but also controls genes involved in CLL pathogenesis such as *MIR155HG*, BCR signaling associated genes, and *CXCR4* [42]. These data suggest that MC2 cells could be targeted by BET inhibitors such as PLX51107. Finally, MC2 cells could be targeted by agents such as metformin in combination or not with venetoclax [43] due to over-expression of genes involved in the mitochondrial activity and metabolism. Altogether MC2 cells can be defined as circulating activated lymph node cells targetable by new therapeutic strategies that will have to be validated both in vitro and in vivo.

As a BTK and ITK inhibitor, it was not surprising that ibrutinib treatment affected both B leukemic cells and immune cells. We defined here an ibrutinib genes signature shared by all cellular subsets which increased under ibrutinib exposure. Some of these genes such as *CXCR4*, *RGS1*, *RGS2* are well known to be involved in CLL pathogenesis and/or lymphocyte migration. But to our knowledge, the fact that ibrutinib up-regulated the same genes in T lymphocytes or NK cells was not known. Targeting CXCR4 is a recent therapeutic option for CLL [44]. Based on our results however, this strategy might not offer a real benefit for patients, since T cells will be depleted. In CLL T lymphocytes exhibit impaired synapse formation and exhaustion features [45, 46]. It has been reported that short ibrutinib treatment (8 weeks) promotes T cell expansion and function in CLL patients [47] while Yin et al. showed that T lymphocytes decrease during ibrutinib therapy [48]. Here, blood monitoring by flow cytometry showed a decrease of all immune cells at M3 post treatment, followed by an increase of CD8 T lymphocytes during progression (M27). Activation status, cytotoxic capacities, and exhaustion were differentially affected by ibrutinib treatment in immune cells but our results strongly suggested that ibrutinib enhanced CD8 cytotoxic capacities even during progressive disease. This observation supports recently published clinical data of CD19-targeted CAR-T cell efficacy [49] or the use of CD19/CD3 bispecific antibody which is highly effective even in ibrutinib-resistant CLL disease [50]. However, our observation showing a decrease in CD19 and an increase in CD20 expression at the surface of B leukemic cells in progressive disease, could be in favor of the use of an CD20/CD3 bispecific antibody [51].

## Conclusions

Dissecting CLL disease using high-dimensional single-cell technologies is fundamental to a better knowledge of biological features contributing to response to therapy and relapse. Our study showed that CITE-Seq approach was a powerful methodology to analyze the CLL disease in its microenvironment context. Our results revealed intra-clonal heterogeneity in B leukemic cells, up-regulation of common signaling pathways induced by ibrutinib and transcriptional and phenotypical modifications in both B leukemic cells and immune cells. These results could open new therapeutic strategies for ibrutinib-resistant patients. Clinical options could be directed to B leukemic cells themselves using venetoclax combined or not with BRD4 inhibitor, or based on immunotherapy using bi-specific antibodies or CAR-T cells.

## Supplementary Information

**Additional file 1: Figure S1. **UMAP representation of integrated CITE-Seq data according to gene expression (mRNA) and cell surface protein (ADT-labeling). **Figure S2.** Ibrutinib regulated pathways in MC2 leukemic cells. **Table S1.** Clinical features before treatment. **Table S2.** Ibrutinib up-regulated genes at relapse. **Table S3.** Gene signatures.

## Data Availability

Data supporting the findings of this work are available within the paper and its Additional Information files. The datasets generated and analyzed during the current study are deposited at NCBI’s Gene Expression Omnibus under accession number GSE152469.

## Abbreviations

1 L: First-line
ADT: Antibody-Derived-Tag
BCR: B cell receptor
BET: Bromodomain and Extra-Terminal motif
BTK: Bruton Tyrosine Kinase
CITE-Seq: Cellular Indexing of Transcriptomes and Epitopes by sequencing
CLL: Chronic Lymphocytic Leukemia
DEGs: Differential Expressed Genes
ITK: IL-2-inducible T cell kinase
MC: Metacells
NK: Natural Killer
NF-κB: Nuclear factor-kappa B
PBMC: Peripheral Blood Mononuclear Cells
PCA: Principal Component Analysis
PLCγ2: 1-Phosphatidylinositol-4,5-bisphosphate phosphodiesterase gamma-2
R/R: Relapsed/refractory
RNA-Seq: RNA sequencing
TEC: Tyrosine-protein kinase Tec
UMAP: Uniform Manifold Approximation and Projection
UMI: Unique Molecular Identifiers
