# Supplementary material for: Longitudinal CITE-Seq profiling of chronic lymphocytic leukemia during ibrutinib treatment: evolution of leukemic and immune cells at relapse
Source: Biomark Res. 2020 Dec 9;8:72. doi: 10.1186/s40364-020-00253-w (PMC7724843; doi:10.1186/s40364-020-00253-w)
Supplement: Supplementary file 1 — Additional file 1: Figure S1. UMAP representation of integrated CITE-Seq data according to gene expression (mRNA) and cell surface protein (ADT-labeling). Figure S2. Ibrutinib regulated pathways in MC2 leukemic cells. Table S1. Clinical features before treatment. Table S2. Ibrutinib up-regulated genes at relapse. Table S3. Gene signatures. [file 40364_2020_253_MOESM1_ESM.pdf]

Longitudinal CITE-Seq profiling of Chronic Lymphocytic Leukemia during ibrutinib treatment: evolution of leukemic and immune cells at relapse.

Cadot *et al*

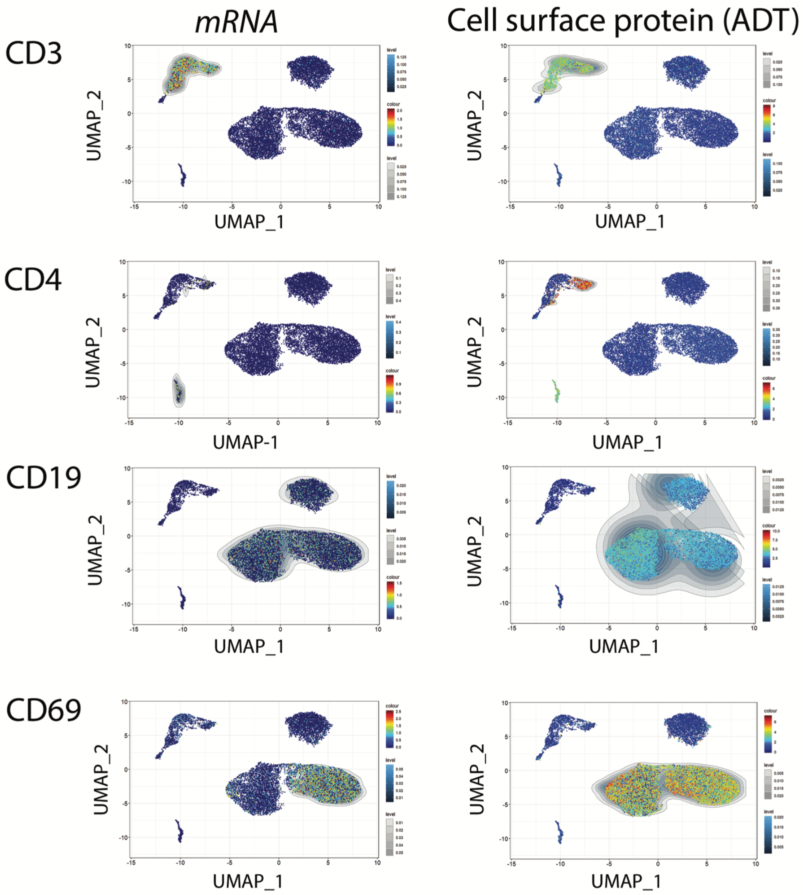

Additional Fig. 1. UMAP representation of integrated CITE-Seq data according to gene expression (mRNA) and cell surface protein (ADT-labeling).

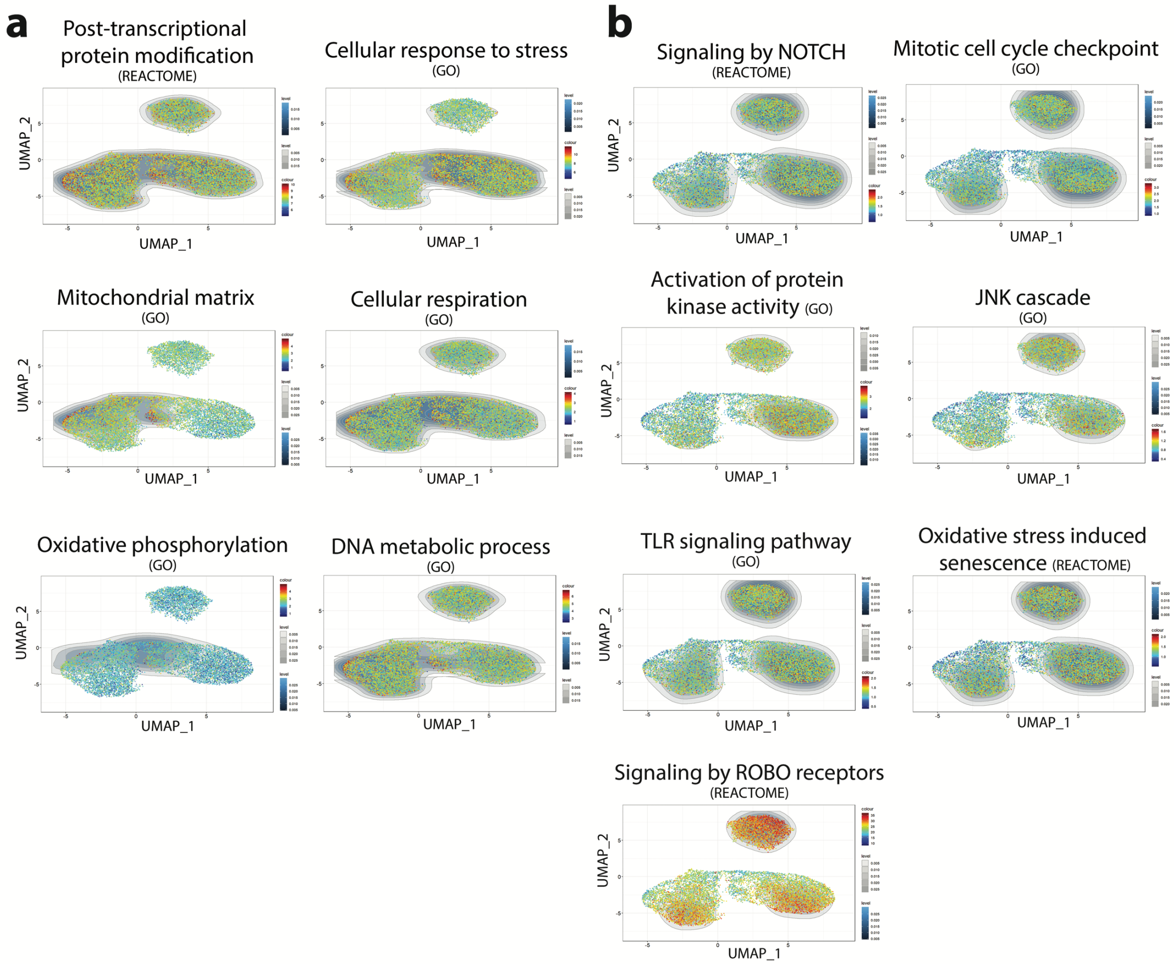

Additional Fig. 2: Ibrutinib regulated pathways in MC2 leukemic cells. **(a)** UMAP representation of differential up-regulated pathways between MC1 and MC2 cells; **(b)** UMAP representation of differential down-regulated pathways between MC1 and MC2 cells.

	Clinical Features
Gender	M
Age	60
IGHV status	UM
Del17p	No
Del11q	No
Del13q	No
Tri12	No
TP53 mutation	No
SF3B1 mutation	No
NOTCH1 mutation	No
Myd88 mutation	No
BTK mutation	No
PLCγ2 mutation	No
Complex karyotype	Yes
Bulky lymph node	No
Previous treatments	>2

Additional Table 1. Clinical features before treatment

Up-regulated genes at relapse
BCL2
BCL2L11
CCL4
CD69
CD70
CNOT1
CTLA4
CXCR4
DDIT4
DUSP1
DUSP10
DUSP4
**DUSP5**
FOSB
JUN
JUND
KIF9
KLF10
KLF6
KLHL15
NFKBIA
PMAIP1
RGS1
RGS16
RGS2
RHOB
STAT4
TOX2
TP53INP2
USP12

Additional Table 2. Ibrutinib up-regulated genes at relapse

Up-regulated genes at relapse (compared to M0, pBH < 0.001, fold change > 4)

Genes in bold represented those up-regulated only in B leukemic cells (compared to Additional Table1)

T cytotoxic signature	T activation signature	T exhaustion signature	BCR Activation[34]		CXCR4 Signaling[33]			Lymph Node signature[34]	NF-kB signature pathway[34]
FCGR3A	CADM1	BTLA	AARS	NAB2	ADRBK1	ITGA11	PXN	AICDA	BCL2A1
FGBP2	CD1D	CD160	ADM2	NAMPT	AKT1	ITGA2	RAC1	AURKA	CCL3
GNLY	CD2	CD244	AKAP1	NFKB1	ARR3	ITGA3	RALB	AURKB	CCL4
GZMA	CD24	CTLA4	ARID5B	NFKBIE	ARRB2	ITGA4	RAP1B	BIRC5	CCND2
GZMB	CD276	HAVCR2	BACH2	NHP2	BAD	ITGA5	RGS1	BUB1B	CD83
GZMH	CD28	LAG3	BCL2	NME1	BCAR1	ITGA6	RHOA	CCL3	CXCL10
GZMK	CD3D	PDCD1	CASP3	NR4A3	BLK	ITGA7	RHOB	CCL4	DUSP2
PRF1	CD3E	TIGIT	CCL4	OAS1	CD247	ITGA8	RHOC	CCNA2	EBI3
	CD4	VSIR	CD58	OAS3	CD3D	ITGA9	RICTOR	CCNB1	GADD45B
	CD47		CD63	PAICS	CD3E	ITGAV	SRC	CCNB2	HEATR1
	CD7		CD72	PEA15	CD3G	ITGB1	SSH1	CCND2	ID2
	CLEC7A		CD83	PIM3	CD4	JAK2	STAT1	CCNL1	JUNB
	CRTAM		CIITA	RGS10	CDC42	LCK	STAT2	CD69	LTA
	EBI3		CLEC2D	SLAMF7	CFL1	LIMK1	STAT3	CD83	NFKBIE
	ELF4		CTLA4	SOCS5	CRK	LYN	STAT5A	CDC6	PBEF1
	FOXP3		DDIT3	SPAG9	CSK	MAPKA	STAT5B	CDT1	RGS1
	GLMN		DDX21	TLK1	CXCL12	P1	UBQLN1	CENPE	TNF
	ICOSLG		DTX1	TNFAIP3	CXCR4	MLST8	VAV1	CHEK1	
	IL12B		DUSP2	TXNRD1	DNM1	MMP9	VPS4A	CR2	
	IL18		EGR1	WARS	FGR	MTOR	VPS4B	CTLA4	
	IL2		EGR3		FOXO1	PAG1	YES1	DUSP1	
	IL21		GARS		FYN	PAK1		DUSP2	
	IL27		GFI1		GNA13	PDPK1		EGR1	
	IL4		GNPDA1		GNAI1	PIK3CA		EGR2	
	IL7		GPX4		GNAI2	PIK3CB		EGR3	
	INS		HLA-		GNAI3	PIK3CD		FOS	
	JAG2		DQA1		GNAO1	PIK3CG		JUN	
	LAT		HLA-		GNAO1	PIK3R1		JUNB	
	LAX1		DQB1		GNAZ	PIK3R2		MCM4	
	LCK		IARS		GNB1	PIK3R3		MCM6	
	NCK1		IFI30		GNB2L1	PIK3R5		MKI67	
	NCK2		IRF4		GNG2	PIK3R6		MYC	
	NHEJ1		ITPKB		GRK6	PLCB1		NAMPT	
	NLRC3		KIAA01		HCK	PLCB2		RGS1	
	PTPRC		KLF10		HGS	PLCB3		TK1	
	SART1		LILRA4		HLA-DRA	PRKCZ		TOP2A	
	SFTPD		LPL		HLA-	PTEN		TTK	
	SIRPG		LRMP		DRB1	PTK2		TYMS	
	SIT1		LY9		INPP5D	PTK2B			
	SLA2		MX1		ITCH	PTPN11			
	SOCS5		MYO1E		ITGA1	PTPN6			
	SPINK5				ITGA10	PTPRC			
	THY1								
	ZAP70								

Additional Table 3. Gene signatures.
